# Brain functional networks reorganization for maintaining gait abilities in aging

**DOI:** 10.18632/aging.204556

**Published:** 2023-03-03

**Authors:** Amgad Droby, Inbal Maidan, Anat Mirelman

**Affiliations:** 1 Laboratory for Early Markers of Neurodegeneration (LEMON), Neurological Institute, Tel Aviv Sourasky Medical Center, Tel Aviv, Israel; 2 Center for Movement, Cognition, and Mobility (CMCM), Neurological Institute, Tel Aviv Sourasky Medical Center, Tel Aviv, Israel; 3 Neurology Department, Sackler Faculty of Medicine, Tel Aviv University, Tel Aviv, Israel; 4 Sagol School for Neuroscience, Tel Aviv University, Tel Aviv, Israel

**Keywords:** aging, brain reserve, brain resilience, gait, functional integration

Neural changes occurring at the molecular and functional levels in the brain as a function of age were reported by numerous studies. These age-related alterations manifest as local structural and metabolic changes and large-scale functional re-organization of neural circuits, and are accompanied by a decline in multiple cognitive and motor skills [1, 2]. However, the aging process is heterogeneous, varying extensively between individuals. A potential explanation for this variance relates to the concepts of brain reserve, cognitive reserve, and their contribution to brain resilience. Brain resilience is a broad term increasingly used to refer to [one’s] capacity to withstand and maintain functions in the face of aging or disease. Brain reserve (BR) is proposed to be one aspect of brain resilience, offering a quantifiable measure of the neurobiological characteristics of the underlying neural tissue (i.e., neuronal density, tissue volumes, etc.), regardless of ongoing degenerative processes in normal aging or other ensuing neurological conditions [3]. Intracranial brain volume (ICV), grey (GM), and white matter (WM) volumes, as well as regional brain volume measures that can be exerted from anatomical MRI scans, are widely used proxies for quantifying BR. Other *in vivo* imaging measures including cortical thickness, microstructural WM measures and positron emission tomography (PET) measures for synaptic integrity have gained interest recently as surrogates for BR assessment [4]. However, reserve is likely a dynamic construct that is also affected by cognitive reserve; the cognitive abilities acquired before the onset of neural deterioration. Thus, structural brain measures might not suffice to fully depict active processes such as functional re-organization of brain circuits. By relying on a local change of the blood oxygenation levels (BOLD signal) providing an indirect measure of neuronal activity, resting-state (rs)-fMRI allows to model the temporal correlation of the measured BOLD signal time course in spatially-distributed, functionally-connected brain regions. Rs-fMRI methods have emerged as sensitive tools for monitoring changes in functional brain networks as a function of aging, disease progression, and treatment outcomes in many neurological and neuropsychiatric conditions. The quantified strength of the connection between regions may reflect mutually activated brain areas revealing various cognitive and motor networks, such as the executive control network (ECN) and sensorimotor network (SMN). Hence, fMRI-based measures might serve as a sensitive, non-invasive mode to characterize neural networks’ spatial and temporal dynamics and explore active neural compensatory mechanisms throughout the life span. A growing mass of evidence from fMRI studies demonstrates reduced intra-network functional (FC) and increased inter-network integration among large-scale functional brain networks in aging [5]. These dynamic changes in the brain, at the functional level throughout the lifespan can have various effects on daily life functioning. Varangis et al. investigated the association between intra-and-inter-network FC of twelve major functional brain networks at rest and cognitive functioning in a large sample of N=665 healthy adults between 20-80 years of age. Corroborating previous findings, the authors of this study reported that age was associated with large-scale re-organization of the neural networks, which included decreased within-network FC and reduced inter-network segregation. These observed functional alterations were also found to be associated with poorer performance in four cognitive domains measuring processing speed, memory, fluid reasoning, and episodic memory. Specifically, greater inter-network segregation correlated with better processing-speed, fluid reasoning, and memory tasks reflecting a more efficient multi-sensory integration. On the other hand, connectivity within the discrete networks was found to be associated with domain-specific cognitive performance [6].

Building on this study, we sought to further explore the relationship between motor-cognitive inter-network connectivity and gait performance as a function of age. It is well established that gait is a complex behavior that relies on multi-level integration between sensory, motor, and higher-order cognitive functions. To this end, we investigated the association between inter-network FC, cognitive performance, and gait performance in both usual (UW) and a cognitively-demanding dual-task (DT) walking conditions as a function of age in N=115 community-dwelling healthy adults within two age categories (young vs. older adults). As expected, UW performance was primarily associated with FC integration between motor and sustained attention networks in the young adults (<65 years) group. On the other hand, in this group, DT walking performance relied on cognitive performance and FC integration between motor and divided attention networks, including the ventral and salience networks, and processing speed cognitive performance.

Interestingly, different patterns were observed in the older group (>65 years). Namely, despite their preserved performance in both walking conditions compared to the younger group, older adults utilized comparable cognitive and neural resources for the simpler walking task (i.e., UW) performance as those observed in younger adults associated with under the more demanding DT walking condition. Interestingly, under DT walking, older adults were observed to rely on FC integration between motor and divided attention networks during the cognitively demanding DT condition. These findings indicate that due to the reduced brain and cognitive reserves as a function of age, older adults utilize greater resources even during simple tasks than younger adults. In addition, older adults allocated more resources to the walking condition at the expense of the cognitive task to maintain uninterrupted walking performance and avoid falls [7].

Cognitive and brain reserve can be seen as dynamic constructs that vary within-and-between individuals under different circumstances. Accumulating evidence from functional imaging studies in aging and neurological conditions strongly suggests that functional brain measures can offer valuable insights into the functional network re-structuring or dedifferentiation dynamics. Multi-modality multi-method approaches combining brain structural and functional measures that reflect active compensatory brain processes may be essential to capture brain reserve under aging effects fully. Furthermore, a more comprehensive characterization of the spatial and temporal dynamics of these functional compensatory processes can provide a deeper understanding of brain resilience and potential strategies to harness these brain capacities for delaying or slowing decline, altering aging trajectories.
